# Predictive Value of Machine Learning for the Risk of In‐Hospital Death in Patients With Heart Failure: A Systematic Review and Meta‐Analysis

**DOI:** 10.1002/clc.70071

**Published:** 2024-12-26

**Authors:** Liyuan Yan, Jinlong Zhang, Le Chen, Zongcheng Zhu, Xiaodong Sheng, Guanqun Zheng, Jiamin Yuan

**Affiliations:** ^1^ Department of Cardiology Affiliated Changshu Hospital of Nantong University Changshu Jiangsu China; ^2^ Department of Cardiology The First People's Hospital of Yancheng, Fourth Affiliated Hospital of Nantong University Yancheng Jiangsu China; ^3^ Department of Cardiology The First Affiliated Hospital of Soochow University Suzhou Jiangsu China

**Keywords:** heart failure, in‐hospital death, machine learning, meta‐analysis, prediction model

## Abstract

**Background:**

The efficiency of machine learning (ML) based predictive models in predicting in‐hospital mortality for heart failure (HF) patients is a topic of debate. In this context, this study's objective is to conduct a meta‐analysis to compare and assess existing prognostic models designed for predicting in‐hospital mortality in HF patients.

**Methods:**

A systematic search of databases was conducted, including PubMed, Embase, Web of Science, and Cochrane Library up to January 2023. To ensure comprehensiveness, we performed an additional search in June 2023. The Prediction Model Risk of Bias Assessment Tool was employed to assess the validity and reliability of ML models.

**Results:**

Our analysis incorporated 28 studies involving a total of 106 predictive models based on 14 different ML techniques. In the training data set, these models showed a combined C‐index of 0.781, sensitivity of 0.56, and specificity of 0.94. In the validation data set, the models exhibited a combined C‐index of 0.758, sensitivity of 0.57, and specificity of 0.84. Logistic regression (LR) was the most frequently used ML algorithm. LR models in the training set had a combined C‐index of 0.795, sensitivity of 0.63, and specificity of 0.85, and these measures for LR models in the validation set were 0.751, 0.66, and 0.79, respectively.

**Conclusions:**

Our study indicates that although ML is increasingly being leveraged to predict in‐hospital mortality for HF patients, the predictive performance remains suboptimal. Although these models have relatively high C‐index and specificity, their ability to predict positive events is limited, as indicated by their low sensitivity.

## Graphical abstract

This systematic review analyzed 106 predictive models from 28 studies utilizing 14 machine learning techniques for predicting in‐hospital mortality in heart failure patients. Models demonstrated good discriminative ability (C‐index: 0.758 in validation) with age, BUN, heart rate, sodium, and creatinine levels as key predictors.

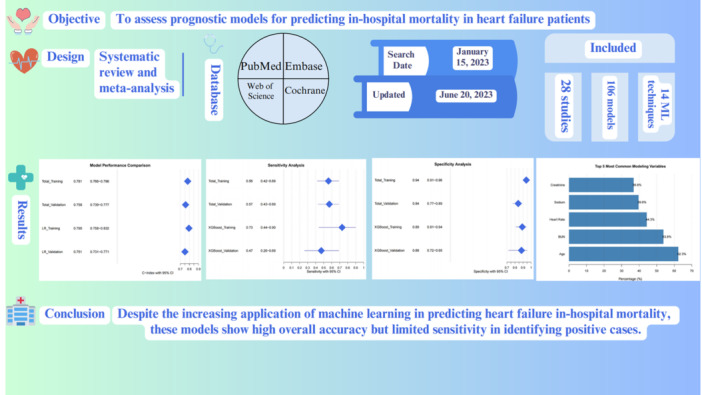

## 1 Introduction

Heart failure (HF) stands as a multifaceted clinical syndrome where symptoms and signs can stem from any structural or functional impairment concerning ventricular filling or ejection [1]. Today, the global population of HF patients continues to burgeon, fueled by an expanding and aging demographic [2]. It is estimated that there are 64.3 million people afflicted with HF worldwide [3]. In developed countries, the known prevalence of HF typically hovers between 1% and 2% among the general adult population [2]. However, the prevalence of “all types” of HF escalates to approximately 11.8% among people aged 65 years and older in developed countries [4]. Interestingly, the prevalence of HF in Asia mirrors that of developed Western countries, resting at around 1% to 1.3% [2]. Comprehensive and population‐based studies to ascertain prevalence and incidence in Northern and Sub‐Saharan Africa have yet to be conducted.

Although drug therapy and device‐based interventions are effective in improving the survival of HF patients [5], their prognosis remains bleak, with a substantially compromised quality of life. Despite the available treatment strategies for HF, many patients continue to suffer with progressive disease [6]. In‐hospital mortality rates for HF remain distressingly high, approaching 5%, and bear a colossal medical expenditure exceeding $100 billion annually [7, 8]. Enhanced triage and stratification of HF patients hold the promise of shaping optimal treatment strategies, especially for high‐risk patients who face a high risk of experiencing adverse events such as hospitalization and death due to worsening HF. This approach assumes a pivotal role in deciding when to implement treatment strategies, ranging from medications and intensified monitoring to advanced care programs and cardiovascular device assistance. Simultaneously, the identification of low‐risk patients has the potential to mitigate patient anxiety and curb the needless squandering of high‐cost medical resources, a facet of paramount importance from the standpoint of health economics [9].

The realm of artificial intelligence (AI) has seamlessly integrated into cardiovascular medicine, sparking a transformative impact across realms such as diagnosis, treatment, risk prediction, drug discovery, and clinical care [10]. At the heart of AI lies machine learning (ML), a domain utilizing models trained on data to make decisions and program algorithms to solve problems. Classification models within ML encompass binary classification, multi‐class classification, multi‐label classification, and imbalanced classification [10]. Delving deeper into the ML spectrum, we encounter deep learning (DL), an advanced branch incorporating more intricate features. DL further branches into artificial neural networks (ANNs), convolutional neural networks, and recurrent DL [10].

Recently, there has been an upsurge in the deployment of ML‐based predictive models aimed at identifying HF patients at heightened risk of in‐hospital mortality. However, the clinical utility of these models remains a contentious topic. Remarkably, no systematic review to date has undertaken the task of consolidating and comparing the performance of these models. To bridge this knowledge gap, we conducted a systematic review and meta‐analysis of models tailored for predicting in‐hospital mortality in HF cases. We delineated their performance metrics and spotlit the most frequently employed predictor variables to furnish a robust reference point for the ongoing evolution and refinement of clinical risk assessment tools.

## 2 Methods

### 2.1 Study Registration

The reporting of this study followed the Preferred Reporting Items for Systematic Reviews and Meta‐Analyses (PRISMA) 2020 guidelines [11]. This study protocol was registered with the International Prospective Register of Systematic Reviews (PROSPERO) with registration number CRD42023408547.

### 2.2 Eligibility Criteria

#### 2.2.1 Inclusion Criteria

1. Inclusion was limited to studies involving patients diagnosed with HF;
2. Studies needed to have developed a complete predictive model for in‐hospital mortality;
3. Studies without external validation were considered;
4. Varying ML studies based on the same data set were eligible for inclusion;
5. The study types encompassed nested case‐control studies, case‐control studies, cohort studies, or case‐cohort studies;
6. We specifically included studies that were reported in English.

#### 2.2.2 Exclusion Criteria

1. Studies falling under categories such as meta‐analyses, reviews, guidelines, expert opinions, and so forth, were excluded;
2. Studies that solely conducted risk factor analyses without constructing a complete ML model were not considered;
3. Exclusion was enforced if studies lacked the following outcome measures to examine the accuracy of ML models, including receiver operating characteristic (ROC) curve, C‐statistic, C‐index, sensitivity, specificity, accuracy, recovery, precision, confusion matrix, calibration curve, diagnostic quartet, and F1 score;
4. Studies solely focused on the accuracy of single‐factor prediction were also excluded.

### 2.3 Data Sources and Search Strategy

A systematic search was conducted to identify relevant studies across databases including PubMed, Embase, Web of Science, and Cochrane Library, spanning up to January 15, 2023. We performed searches based on subject headings and keywords, with no geographical restrictions applied (Supporting Information S1: Table 1 for details). To mitigate the risk of missing articles, we conducted an additional search on June 20, 2023.

### 2.4 Study Selection and Data Extraction

Following the retrieval of literature, we imported it into EndNote to remove duplicates and initially screened the studies based on their titles and abstracts, where studies failing to meet the predefined eligibility criteria were removed. Subsequently, we accessed and examined the remaining full‐text studies, subjecting them to a rigorous assessment against our predefined eligibility criteria. In preparation for data extraction, we developed a table including items such as title, first author, publication year, country, subject source, total case count, number of recorded death cases, total cases in the training set, death cases in the training set, methodology used to generate the validation set, steps taken to minimize overfitting, total cases within the validation set, death cases within the validation set, handling of missing values, variable selection methodology, model type, and variables incorporated within the model.

Study selection and data extraction were independently carried out by two investigators (Liyuan Yan and Guanqun Zheng) with cross‐verification. Any discrepancies were resolved through discussion with a third investigator (Xiaodong Sheng) until consensus was reached.

### 2.5 Risk of Bias in Studies

The Prediction Model Risk of Bias Assessment Tool (PROBAST) was utilized to assess the potential bias within the original studies included in our analysis. PROBAST consists of a set of questions distributed across four different domains. These domains collectively provide a comprehensive overview of the risk of bias and the overall quality of studies. These domains pertain to participants, predictors, results, and statistical analyses [12]. Each domain contains a varying number of specific questions—2, 3, 6, and 9, respectively. Respondents had three options for each question: “yes/maybe,” “no/may not,” and “no information.” A study evaluated for a domain was considered at a high risk of bias if this domain had at least one question receiving a “no” or “probably no” response. Conversely, to be classified as low risk, all questions within a domain needed to receive a “yes” or “possibly yes” response. The overall risk of bias was considered low when all domains were rated as low risk and high when at least one domain was categorized as high risk. Quality assessment was independently conducted by two researchers (Liyuan Yan and Le Chen), who subsequently cross‐verified their evaluations. Any discrepancies were resolved through discussion with a third researcher (Xiaodong Sheng).

### 2.6 Outcomes

Our systematic review primarily focused on three primary outcomes: the C‐index, sensitivity, and specificity. Additionally, we conducted an extensive examination of the variables incorporated into ML‐based models.

### 2.7 Synthesis Methods

The performance of ML models across included studies was evaluated through a meta‐analysis of three metrics: C‐index, sensitivity, and specificity. In cases where 95% confidence intervals (CIs) and standard errors of the C‐index were not available, we estimated standard errors using a reference method outlined by Debray et al. [13]. Given the diversity in variables included in the ML models and the variability in parameters, we opted for a random‐effects model for the meta‐analysis of the C‐index, and we performed a meta‐analysis of sensitivity and specificity using a bivariate mixed‐effects model. We further conducted subgroup analyses based on the data set (training set or validation set) and the type of model employed. During the meta‐analysis process, we analyzed sensitivity and specificity based on the diagnostic quartet table. However, most original studies did not report the diagnostic quartet table. In such instances, we calculated the diagnostic quartet table through two approaches: 1. By combining the number of cases to compute sensitivity, specificity, and precision. 2. By extracting sensitivity and specificity based on the best Youden index, followed by subsequent calculations based on case numbers. The meta‐analysis was performed in STATA 15.0 (Stata Corp LP, College Station, TX).

## 3 Results

### 3.1 Study Selection

**Figure 1:**
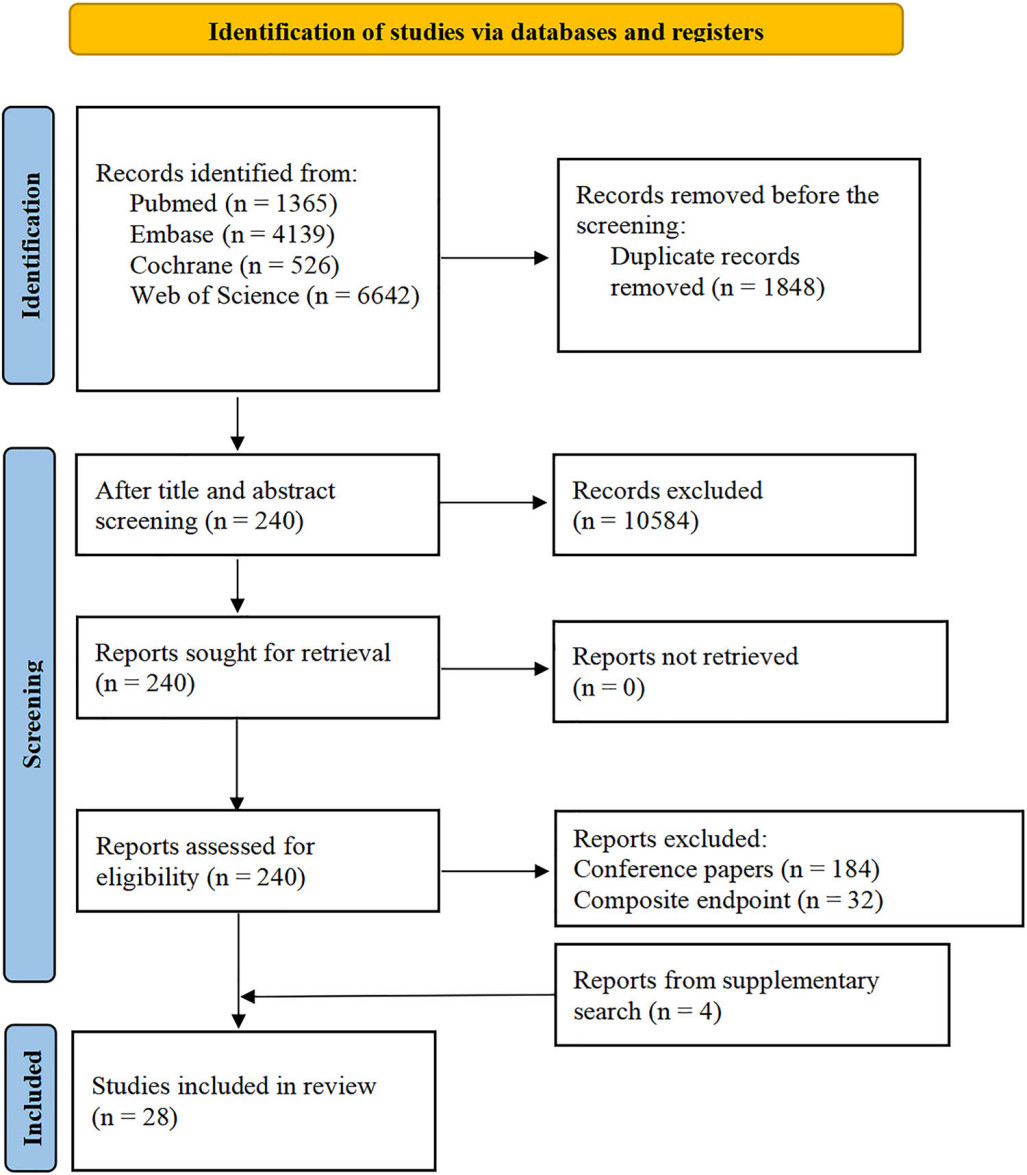
PRISMA (Preferred Reporting Items for Systematic Reviews and Meta‐Analyses) flow diagram for study selection.

Following the initial search, we identified 12 672 articles. Upon removing 1848 duplicates, we were left with 10 824 publications for further scrutiny through title and abstract screening. Out of these, 240 articles met the criteria for full‐text screening, and 184 of them were excluded because they were conference papers with limited information, particularly in terms of methodology and risk of bias. The remaining 56 underwent a comprehensive full‐text assessment and 32 of them were removed due to their reliance on composite endpoints. Ultimately, we retained 24 studies and added 4 studies retrieved from the supplemental search for data extraction [14, 15, 16, 17, 18, 19, 20, 21, 22, 23, 24, 25, 26, 27, 28, 29, 30, 31, 32, 33, 34, 35, 36, 37, 38, 39, 40, 41]. Figure 1 presents our study selection process as per the PRISMA guidelines.

### 3.2 Study Characteristics

Supporting Information S1: Table 2 summarizes the basic characteristics of the 28 included studies. These studies span from the year 2008 to 2023, with a notable surge in publications, as 24 out of the 28 studies (85.7%) were published after 2020. The data set comprises studies from six countries, including China (19), the United States (4), Japan (2), South Africa (1), South Korea (1), and Germany (1). Among these, 21 studies drew their data from registries, prospective cohort studies, or nested case‐control studies, while 7 studies drew their data from retrospective cohort studies. Eighteen of the 28 studies developed two or more distinct models. The top three most frequently employed modeling approaches were logistic regression (LR, 92.9%), random forest (RF, 46.4%), and eXtreme Gradient Boosting (XGBoost, 42.9%). As for validation, 23 studies (82.1%) subjected their models to internal validation through methods such as random sampling method, K‐fold cross‐validation, or bootstrapping. Only 11 out of the 28 studies (39.3%) utilized external validation.

### 3.3 Risk of Bias in Studies

**Figure 2:**
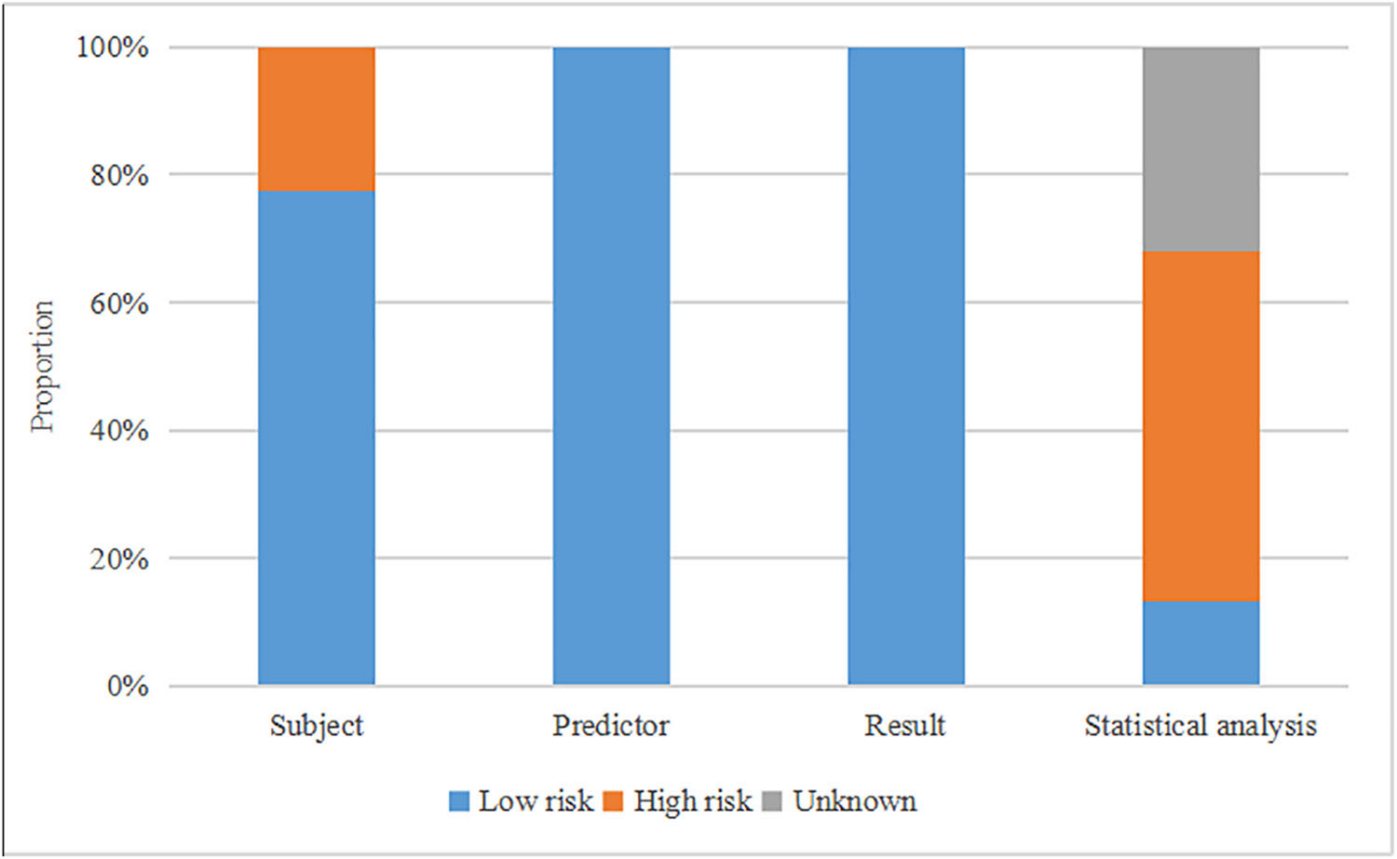
Risk of bias assessment with PROBAST (Prediction Model Risk of Bias Assessment Tool) of 28 meta‐analyzed studies containing 106 models.

Figure 2 provides a comprehensive overview of the risk of bias assessments conducted across the 28 studies. This assessment encompassed individual risk of bias evaluations for each model, resulting in 106 assessments in total. Notably, 82 of these models (77.4%) demonstrated a low risk of subject bias, while the remaining 24 (22.6%) exhibited a high risk of subject bias. Predictor bias and result bias were consistently rated as low risk across all 106 models. However, the assessment of statistical analysis bias revealed that 14 models (13.2%) presented a low risk, 34 models (32.1%) held an unknown risk of statistical analysis bias, and 58 models (54.7%) indicated a high risk. In summary, considering the overall risk of bias, 14 models (13.2%) were categorized as having a low risk of bias, 24 (22.6%) had an unknown risk, and 68 (64.2%) were designated as having a high risk.

### 3.4 Meta‐Analysis

#### 3.4.1 C‐Index

Table 1 presents the pooled C‐index for various models based on different ML techniques, of which 54 models underwent external validation. The training set encompassed 90 models, involving 895 024 samples in total. The overall pooled C‐index (95% CI) of these training set models was 0.781 (0.766, 0.796). In the training set, the most frequently used ML methods included LR, support vector machine (SVM), RF, light gradient boosting machine (LightGBM), XGBoost, and ANN. Notably, LR was utilized in 21 predictive and exhibited a pooled C‐index (95% CI) of 0.795 (0.758, 0.832) for predicting in‐hospital mortality in HF patients. The pooled C‐index (95% CI) for SVM, RF, LightGBM, XGBoost, and ANN was 0.755 (0.650, 0.859), 0.805 (0.768, 0.842), 0.818 (0.794, 0.841), 0.831 (0.762, 0.899), and 0.775 (0.683, 0.867), respectively. Among these diverse ML methods, XGBoost exhibited the highest pooled C‐index in the training set.

**Table 1:** Summary of C‐index of models based on different machine learning methods.

Model types	Training set			Validation set		
	sample size	*n*	c‐index (95%CI)	sample size	*n*	c‐index (95%CI)
SVM	93104	13	0.755 (0.650−0.859)	7349	2	0.625 (0.432−0.818)
DT	9991	4	0.662 (0.587−0.737)	2590	1	0.685 (0.660−0.710)
RF	102919	10	0.805 (0.768−0.842)	663289	11	0.788 (0.761−0.814)
LightGBM	83564	10	0.818 (0.794−0.841)	17371	2	0.835 (0.743−0.928)
LR	341551	21	0.795 (0.758−0.832)	264354	20	0.751 (0.731−0.771)
Bagging	7740	2	0.728 (0.542−0.914)	2590	1	0.736 (0.713−0.759)
KNN	7740	2	0.621 (0.550−0.693)	2590	1	0.616 (0.590−0.642)
XGBoost	104838	10	0.831 (0.762−0.899)	30307	6	0.809 (0.769−0.849)
DL	28091	5	0.803 (0.719−0.887)	4759	1	0.880 (0.876−0.884)
ANN	99130	9	0.775 (0.683−0.867)	18755	4	0.745 (0.602−0.888)
NB	7296	2	0.661 (0.560−0.762)	3282	2	0.681 (0.548−0.815)
CatBoost	4530	1	0.821 (0.806−0.836)	2590	1	0.806 (0.786−0.826)
AdaBoost	4530	1	0.796 (0.780−0.812)	2590	1	0.714 (0.690−0.738)
BNN	/	/	/	4759	1	0.730 (0.721−0.739)
Overall	895024	90	0.781 (0.766−0.796)	1027175	54	0.758 (0.739−0.777)

Abbreviations: AdaBoost, adaptive boosting; ANN, artificial neural network; BNN, Bayesian network; CatBoost, categorical boosting; CI, confidence interval; DL, deep learning; DT, decision tree; KNN, K‐nearest neighbors; LightGBM, light gradient boosting machine; LR, logistic regression; NB, Naive Bayes; RF, random forests; SVM, support vector machine; XGBoost, eXtreme Gradient Boosting.

Moving to the validation set, which encompassed 54 models involving a total of 1 027 175 samples. The overall pooled C‐index (95% CI) for these models was 0.758 (0.739, 0.777). In the validation set, LR‐based models (20 in total) yielded a pooled C‐index (95% CI) of 0.751 (0.731, 0.771) for predicting in‐hospital mortality in HF patients. Eleven models based on RF produced a pooled C‐index (95% CI) of 0.788 (0.761, 0.814), and six XGBoost‐based models achieved a pooled C‐index (95% CI) of 0.809 (0.769−0.849) for predicting in‐hospital mortality in HF patients.

#### 3.4.2 Sensitivity and Specificity

Table 2 shows the pooled sensitivity and specificity across different ML methods. Only 59 models in the training set provided enough information for sensitivity and specificity calculations, and only 22 models in the validation set provided enough information. In the training set, the overall pooled sensitivity (95% CI) and specificity (95% CI) for all models were 0.56 (0.42, 0.69) and 0.94 (0.91, 0.96), respectively. Notably, XGBoost, encompassing seven models, achieved the highest sensitivity (95% CI) of 0.73 (0.44, 0.90) among all ML methods. Moreover, all ML methods incorporating over three models demonstrated high specificity (95% CI) of 0.93 (0.81, 0.97), 0.97 (0.96, 0.98), 0.94 (0.86, 0.98), 0.85 (0.75, 0.92), 0.89 (0.81, 0.94), 0.95 (0.90, 0.98) and 0.91 (0.85, 0.97) for SVM, RF, LightGBM, LR, XGBoost, DL, and ANN, respectively.

**Table 2:** Summary of sensitivity and specificity of models based on different machine learning methods.

Model types	Training set				Validation set			
	Sample size	*n*	Sensitivity (95% CI)	Specificity (95% CI)	Sample size	*n*	Sensitivity (95%CI)	Specificity (95%CI)
SVM	85 014	10	0.49 (0.18−0.80)	0.93 (0.81−0.97)	150	1	0.430	0.944
DT	2251	2	0.568−0.940	0.845−0.894	150	1	0.700	0.792
RF	55 365	8	0.37 (0−0.90)	0.97 (0.96−0.98)	6157	2	0.600−0.713	0.6640−0.6642
LightGBM	28 270	6	0.59 (0.36−0.79)	0.94 (0.86−0.98)				
LR	86 902	15	0.63 (0.46−0.77)	0.85 (0.75−0.92)	33142	11	0.66 (0.52−0.78)	0.79 (0.71−0.86)
XGBoost	52 754	7	0.73 (0.44−0.90)	0.89 (0.81−0.94)	11737	4	0.47 (0.26−0.69)	0.88 (0.72−0.95)
DL	28 091	5	0.41 (0.19−0.67)	0.95 (0.90−0.98)	4759	1	0.832	0.771
ANN	47140	5	0.39 (0−0.95)	0.91 (0.85−0.97)	692	1	0.028	0.988
NB	2766	1	0.114	0.972	692	1	0.165	0.963
Overall	388 553	59	0.56 (0.42−0.69)	0.94 (0.91−0.96)	57479	22	0.57 (0.43−0.69)	0.84 (0.77−0.89)

Abbreviations: ANN, artificial neural network; CI, confidence interval; DL, deep learning; DT, decision tree; LightGBM, light gradient boosting machine; LR, logistic regression; NB, Naive Bayes; RF, random forests; SVM, support vector machine; XGBoost, eXtreme Gradient Boosting.

In the validation set, only LR and XGBoost included more than three models. The pooled sensitivity (95% CI) and specificity (95% CI) for LR were 0.66 (0.52, 0.78) and 0.79 (0.71, 0.86), respectively. For XGBoost, the pooled sensitivity (95% CI) and specificity (95% CI) were 0.47 (0.26, 0.69) and 0.88 (0.72, 0.95), respectively.

### 3.5 Variables Used for Modeling

Table 3 displays the 15 most frequently employed features within prognostic models. The top five most commonly integrated variables across all models were age (62.3%), blood urea nitrogen (BUN, 53.8%), heart rate (44.3%), sodium levels (39.6%), and creatinine levels (36.8%). In the context of intensive care unit‐based (ICU‐based) HF patient prognosis models, Supporting Information S1: Table 3 summarizes the 10 most frequently used modeling variables. Within this specialized subset, the five most prevalent modeling variables were age (82.6%), BUN (67.4%), urine output (60.9%), N‐terminal pro‐B‐type natriuretic peptide (NT‐proBNP, 58.7%), and mechanical ventilation (58.7%).

**Table 3:** The most frequent factors included in risk prediction models for in‐hospital death in patients with heart failure.

Studies	Age	BUN	HR	Sodium	Creatinine	Gender	NT‐proBNP	Respiratory rate	SBP	Albumin	Platelets	WBC	Acute kidney injury	Potassium	Urine output
Yang [14]					6	6					6		6		
Yang [15]	9	9	9				9	9	9	9					9
Yan [16]	1				1										
Wang [17]		8	8	8											
Wang [18]		5	5	5											
Wang [19]	1	1		1			1		1			1			
Wang [20]					5					5	5			5	
Shiraishi [21]	1	1		1			1		1	1					
Segar [22]	5	5	5	5	5		5	5	5					5	
Peng [23]	5	5	5				5	5				5			
Lv [24]	5			5	5	5		5	5	5		5			
Luo [25]	2	2			2			2			2				2
Li [26]	11						11						11		11
Li [27]	4	4	4			4		4	4		4	4	4		4
Li [28]	2	2	2	2			2	2			2	2		2	2
Lagu [29]	1					1							1		
Kwon [30]	5	5	5	5	5	5			5	5	5	5		5	
König [31]	5					5									
Jia [32]			1				1								
Han [33]	1	1	1		1			1	1			1			
Gao [34]	1														
Dai [35]	3					3									
Chen [36]	1		1	1					1			1	1		
Abraham [37]	1		1	1	1				1						
Chen [38]	2							2							
Ma [39]		8		8	8	8				8	8	8	8	8	
Misumi [40]		1							1						
Mpanya [41]														6	
Total	66	57	47	42	39	37	35	35	34	33	32	32	31	31	28

*Note:* The numbers in the table (such as 6's and 9's) represent the frequency of variable usage across different prediction models within the same study. For instance, Jie Yang, et al. developed 6 prediction models using various modeling approaches. Creatinine and gender were used as predictors in all 6 models. This is why you see the number “6” for these variables. The numbers demonstrate how frequently each variable was selected across different modeling methods within the same study.

Abbreviations: BUN, blood urea nitrogen; HR, heart rate; NT‐proBNP, N‐terminal pro‐B‐type natriuretic peptide; SBP, systolic blood pressure; WBC, white blood cell.

## 4 Discussion

### 4.1 Summary of the Main Findings

This review comprehensively evaluates the performance metrics, including the C‐index, sensitivity, and specificity, across a total of 106 predictive models for assessing in‐hospital mortality risk in HF patients, drawing upon outcomes from 28 studies. Furthermore, it highlights the preeminent modeling variables widely adopted within these models. Notably, among the 106 models assessed, only 14 were found to exhibit a low risk of overall bias, 24 presented an unknown risk, while the remaining 68 were categorized as having a high overall risk of bias. Within the landscape of ML methods, LR and XGBoost emerged as the two most frequently utilized techniques. These models, grounded in LR and XGBoost, consistently demonstrated commendable C‐index values and high specificity. However, the predictive models based on various ML methods exhibited a notable shortfall in sensitivity. Across all the studies reviewed, the top five most frequently used modeling variables were age, BUN, heart rate, sodium levels, and creatinine levels. On the other hand, for studies specifically focused on ICU patients grappling with HF, the five most frequently used modeling variables were age, BUN, urine output, mechanical ventilation, and NT‐proBNP.

### 4.2 Comparison with Other Reviews

Many risk prediction models based on different methodologies, including ML, are instrumental in estimating the prognosis of symptomatic HF. Currently, multiple reviews have been published on predictive models for HF outcomes [42, 43, 44]. In 2014, Ouwerkerk et al. conducted a comprehensive meta‐analysis, aggregating data from 117 HF prognostic models derived from 55 studies published worldwide [42]. Their study identified key predictors, with BUN and blood sodium levels emerging as strong indicators of HF. Furthermore, they observed a significant positive correlation between the number of modeling variables included in the final model and the C‐statistic [42]. Interestingly, our study aligns with these findings as BUN and blood sodium levels were among the top five most commonly used predictors, with BUN being the primary predictor in ICU patients. In contrast to Ouwerkerk et al., who reported a mean C‐statistic of 0.66 for all models and 0.68, 0.71, and 0.63 for models predicting HF hospitalization, mortality, or both, respectively [42], our study exhibited higher pooled C‐index values for all models (0.781 in the validation set and 0.758 in the training set). This divergence may be attributed to several factors. First, our study focused solely on in‐hospital mortality, whereas Ouwerkerk et al. encompassed death occurrences overall time frames, with predictive models exhibiting enhanced performance for short‐term outcomes. Second, the studies incorporated into our analysis utilized more advanced ML techniques, potentially contributing to improved predictive accuracy.

Rahimi et al. performed a meta‐analysis involving 64 primary models and 50 modifications drawn from 48 studies [43]. Notably, 43 of the 64 primary models were used to predict death, yielding C‐statistic values ranging from 0.60 to 0.89 [43]. In contrast, our study showcased a wider range of C‐statistic values, spanning from 0.524 to 1.000 for all models, 0.524 to 1.000 for the training set, and 0.526 to 0.899 for the validation set. Rahimi et al. reported a median of 9 predictors included in the primary model, with age, blood pressure, renal function, sodium levels, ejection fraction, gender, New York Heart Association functional class, NT‐proBNP levels, diabetes, exercise capacity, and weight/body mass index emerging as the most commonly frequently used and influential predictors [43]. Our study mirrored their findings, with the 10 most commonly used predictors comprising age, BUN, heart rate, sodium levels, creatinine levels, gender, NT‐proBNP, respiratory rate, systolic blood pressure, and albumin levels.

Shin et al. conducted a meta‐analysis, consolidating data from 20 studies involving 686 842 patients [44]. In their study, tree‐type ML algorithms were the most frequently employed ML technique, while LR dominated traditional statistical methods [44]. Correspondingly, our study demonstrated a parallel preference for tree‐based ML algorithms and LR as the most commonly used modeling methods. Notably, Shin et al. found that, across seven of the nine time points ranging from hospital mortality to 1‐year survival, ML methods outperformed conventional statistical models [44]. In our study, LR‐based models demonstrated robust discrimination in both the training and validation sets, yielding pooled C‐statistic values of 0.795 and 0.751, respectively. Intriguingly, several ML methods surpassed LR‐based models in terms of C‐statistic, including RF, LightGBM, XGBoost, and DL.

In our systematic review, many of the studies we examined developed multiple predictive models, resulting in a total of 106 models derived from 28 different studies. It's essential to underscore the significance of predictive models in clinical applications from two distinct perspectives: model explainability and predictive performance [45]. Some models, such as LR and decision trees, are characterized by high explainability, yet they often exhibit lower predictive accuracy [45]. Conversely, models like XGBoost, SVM, neural network, and DL tend to possess low explainability but demonstrate remarkably high prediction accuracy [45]. However, in our study, we found that regardless of the specific models employed, even those boasting high C‐index values primarily excelled in predicting negative events (i.e., survival). Conversely, the predictive accuracy of these models for positive events (i.e., death) remained lower. This analytical challenge stems from the characteristic distribution of outcomes in HF populations, where acute in‐hospital mortality events, though clinically significant, represent a smaller proportion of the total patient cohort, creating an inherent statistical imbalance in outcome assessment. Regrettably, most current studies do not adequately account for the effects of data imbalance. Therefore, the predictive model constructed in the present stage of research may not be readily applicable in clinical practice for effectively identifying patients with HF at a high risk of in‐hospital death. As we look ahead, future research endeavors should prioritize improving the predictive accuracy concerning positive events (i.e., in‐hospital deaths) by addressing the challenges posed by imbalance.

### 4.3 Advantages and Limitations of the Study

Compared with previous studies, our study offers several advantages. First, it represents the inaugural meta‐analysis encompassing comprehensive data on all ML‐based models for predicting in‐hospital mortality in HF patients. Diverging from previous studies that amalgamated time points, our analysis exclusively focused on summarizing prediction models for in‐hospital death. This refined approach holds particular significance in the context of risk stratification of hospitalized HF patients. Enhanced precision in identifying high‐risk patients can significantly aid clinicians in making informed decisions regarding the implementation of palliative care strategies, including medication regimens, intensified monitoring, advanced care plans, and cardiovascular interventions. Simultaneously, recognizing low‐risk patients alleviates their anxiety and minimizes unnecessary healthcare resource allocation. However, despite the propensity of many ML‐based models to exhibit high specificity, indicative of their efficacy in discriminating negative events, none of these models demonstrate correspondingly high pooled sensitivity, which underscores their limited discrimination capability in predicting positive events. Thirdly, this study breaks new ground by presenting a summary of predictors for ICU patients with HF, providing valuable insights for future model modeling for ICU patients.

Nonetheless, our study does come with certain limitations. First, the scarcity of studies characterized by a low risk of bias is notable, with only 14 out of 106 models meeting these criteria. Second, the lack of sufficient data in many studies hindered our ability to calculate the sensitivity and specificity for each model, potentially impacting the reliability of our analysis results.

## 5 Conclusions

In summary, the paucity of studies exhibiting a low overall risk of bias raises a critical concern among all high‐quality studies. LR currently emerges as the most widely adopted ML method, delivering robust discrimination for negative events (patient survival) but falling short when it comes to positive events (in‐hospital death). It is imperative for future research endeavors to prioritize both quality assessment and the predictive capacity concerning positive events in the development of predictive models for HF patients.

## Author Contributions

Liyuan Yan and Guanqun Zheng conceived the idea of the study. Liyuan Yan, Guanqun Zheng, and Xiaodong Sheng screened the studies and reviewed the selected articles. Liyuan Yan, Guanqun Zheng, and Xiaodong Sheng undertook data extraction. Liyuan Yan carried out the statistical analysis. Guanqun Zheng supervised the analysis. Liyuan Yan, Le Chen, and Zongcheng Zhu interpreted the findings, and Liyuan Yan drafted the manuscript. Liyuan Yan, Guanqun Zheng, Xiaodong Sheng, Le Chen, and Zongcheng Zhu critically reviewed the manuscript, and Liyuan Yan revised the manuscript for final submission. Jinlong Zhang and Jiamin Yuan provided significant assistance during the revision of the manuscript. All authors have approved the final draft of the manuscript. Jiamin Yuan is the guarantor. Jiamin Yuan accepted full responsibility for the work and the conduct of the study, had access to the data, and controlled the decision to publish. Liyuan Yan and Jinlong Zhang should be considered joint first authors. The corresponding author attests that all listed authors meet authorship criteria and that no others meeting the criteria have been omitted.

## Ethics Statement

All analyses were based on previous published studies, thus no ethical approval and patient consent are required.

## Conflicts of Interest

The authors declare no conflicts of interest.

## Supporting information

Supporting information.

## Data Availability

The data that support the findings of this study are available from the corresponding author upon reasonable request.
