# Supplementary material for: Predictive Value of Machine Learning for the Risk of In‐Hospital Death in Patients With Heart Failure: A Systematic Review and Meta‐Analysis
Source: Clin Cardiol. 2024 Dec 26;48(1):e70071. doi: 10.1002/clc.70071 (PMC11670054; doi:10.1002/clc.70071)
Supplement: Supplementary file 1 — Supporting information. [file CLC-48-e70071-s001.docx]

**Supplemental Material**

Supplemental Table S1: Literature search strategy

- 1. **PubMed**

| Search number | Query | Results |
| --- | --- | --- |
| #1 | "Heart Failure"[Mesh] | 143,360 |
| #2 | ((((((((((((((((Heart Failure[Title/Abstract]) OR (Cardiac Failure[Title/Abstract])) OR (Heart Decompensation[Title/Abstract])) OR (Myocardial Failure[Title/Abstract])) OR (myocardial insufficiency[Title/Abstract])) OR (cardiac backward failure[Title/Abstract])) OR (cardiac decompensation[Title/Abstract])) OR (cardiac incompetence[Title/Abstract])) OR (cardiac insufficiency[Title/Abstract])) OR (cardiac stand still[Title/Abstract])) OR (cardial decompensation[Title/Abstract])) OR (cardial insufficiency[Title/Abstract])) OR (decompensatio cordis[Title/Abstract])) OR (heart backward failure[Title/Abstract])) OR (heart incompetence[Title/Abstract])) OR (heart insufficiency[Title/Abstract])) OR (insufficientia cardis[Title/Abstract]) | 227,699 |
| #3 | ("Heart Failure"[Mesh]) OR (((((((((((((((((Heart Failure[Title/Abstract]) OR (Cardiac Failure[Title/Abstract])) OR (Heart Decompensation[Title/Abstract])) OR (Myocardial Failure[Title/Abstract])) OR (myocardial insufficiency[Title/Abstract])) OR (cardiac backward failure[Title/Abstract])) OR (cardiac decompensation[Title/Abstract])) OR (cardiac incompetence[Title/Abstract])) OR (cardiac insufficiency[Title/Abstract])) OR (cardiac stand still[Title/Abstract])) OR (cardial decompensation[Title/Abstract])) OR (cardial insufficiency[Title/Abstract])) OR (decompensatio cordis[Title/Abstract])) OR (heart backward failure[Title/Abstract])) OR (heart incompetence[Title/Abstract])) OR (heart insufficiency[Title/Abstract])) OR (insufficientia cardis[Title/Abstract])) | 260,044 |
| #4 | "Machine Learning"[Mesh] | 53,027 |
| #5 | ((((((((((((((((((((((((machine learning[Title/Abstract]) OR (Transfer Learning[Title/Abstract])) OR (Deep learning[Title/Abstract])) OR (Ensemble Learning[Title/Abstract])) OR (artificial intelligence[Title/Abstract])) OR (Prediction model[Title/Abstract])) OR (random forest[Title/Abstract])) OR (neural network[Title/Abstract])) OR (neural networks[Title/Abstract])) OR (CNN[Title/Abstract])) OR (Support vector machine[Title/Abstract])) OR (SVM[Title/Abstract])) OR (Gradient Boosting Machine[Title/Abstract])) OR (GBM[Title/Abstract])) OR (Nomogram[Title/Abstract])) OR (XGBoost[Title/Abstract])) OR (Adaboost[Title/Abstract])) OR (Decision tree[Title/Abstract])) OR (ResNet-50[Title/Abstract])) OR (ResNet[Title/Abstract])) OR (Fine-Gray[Title/Abstract])) OR (Competitive Risk Model[Title/Abstract])) OR (Naive Bayesian[Title/Abstract])) OR (Risk Prediction[Title/Abstract])) OR (Risk-Prediction[Title/Abstract]) | 274,722 |
| #6 | ("Machine Learning"[Mesh]) OR (((((((((((((((((((((((((machine learning[Title/Abstract]) OR (Transfer Learning[Title/Abstract])) OR (Deep learning[Title/Abstract])) OR (Ensemble Learning[Title/Abstract])) OR (artificial intelligence[Title/Abstract])) OR (Prediction model[Title/Abstract])) OR (random forest[Title/Abstract])) OR (neural network[Title/Abstract])) OR (neural networks[Title/Abstract])) OR (CNN[Title/Abstract])) OR (Support vector machine[Title/Abstract])) OR (SVM[Title/Abstract])) OR (Gradient Boosting Machine[Title/Abstract])) OR (GBM[Title/Abstract])) OR (Nomogram[Title/Abstract])) OR (XGBoost[Title/Abstract])) OR (Adaboost[Title/Abstract])) OR (Decision tree[Title/Abstract])) OR (ResNet-50[Title/Abstract])) OR (ResNet[Title/Abstract])) OR (Fine-Gray[Title/Abstract])) OR (Competitive Risk Model[Title/Abstract])) OR (Naive Bayesian[Title/Abstract])) OR (Risk Prediction[Title/Abstract])) OR (Risk-Prediction[Title/Abstract])) | 279,748 |
| #7 | "Mortality"[Mesh] | 421,219 |
| #8 | (((((((Mortality[Title/Abstract]) OR (Mortalities[Title/Abstract])) OR (Case Fatality Rate[Title/Abstract])) OR (Case Fatality Rates[Title/Abstract])) OR (Death Rate[Title/Abstract])) OR (Death Rates[Title/Abstract])) OR (Survival[Title/Abstract])) OR (Death[Title/Abstract]) | 2,514,008 |
| #9 | ("Mortality"[Mesh]) OR ((((((((Mortality[Title/Abstract]) OR (Mortalities[Title/Abstract])) OR (Case Fatality Rate[Title/Abstract])) OR (Case Fatality Rates[Title/Abstract])) OR (Death Rate[Title/Abstract])) OR (Death Rates[Title/Abstract])) OR (Survival[Title/Abstract])) OR (Death[Title/Abstract])) | 2,643,036 |
| #10 | ((("Heart Failure"[Mesh]) OR (((((((((((((((((Heart Failure[Title/Abstract]) OR (Cardiac Failure[Title/Abstract])) OR (Heart Decompensation[Title/Abstract])) OR (Myocardial Failure[Title/Abstract])) OR (myocardial insufficiency[Title/Abstract])) OR (cardiac backward failure[Title/Abstract])) OR (cardiac decompensation[Title/Abstract])) OR (cardiac incompetence[Title/Abstract])) OR (cardiac insufficiency[Title/Abstract])) OR (cardiac stand still[Title/Abstract])) OR (cardial decompensation[Title/Abstract])) OR (cardial insufficiency[Title/Abstract])) OR (decompensatio cordis[Title/Abstract])) OR (heart backward failure[Title/Abstract])) OR (heart incompetence[Title/Abstract])) OR (heart insufficiency[Title/Abstract])) OR (insufficientia cardis[Title/Abstract]))) AND (("Machine Learning"[Mesh]) OR (((((((((((((((((((((((((machine learning[Title/Abstract]) OR (Transfer Learning[Title/Abstract])) OR (Deep learning[Title/Abstract])) OR (Ensemble Learning[Title/Abstract])) OR (artificial intelligence[Title/Abstract])) OR (Prediction model[Title/Abstract])) OR (random forest[Title/Abstract])) OR (neural network[Title/Abstract])) OR (neural networks[Title/Abstract])) OR (CNN[Title/Abstract])) OR (Support vector machine[Title/Abstract])) OR (SVM[Title/Abstract])) OR (Gradient Boosting Machine[Title/Abstract])) OR (GBM[Title/Abstract])) OR (Nomogram[Title/Abstract])) OR (XGBoost[Title/Abstract])) OR (Adaboost[Title/Abstract])) OR (Decision tree[Title/Abstract])) OR (ResNet-50[Title/Abstract])) OR (ResNet[Title/Abstract])) OR (Fine-Gray[Title/Abstract])) OR (Competitive Risk Model[Title/Abstract])) OR (Naive Bayesian[Title/Abstract])) OR (Risk Prediction[Title/Abstract])) OR (Risk-Prediction[Title/Abstract])))) AND (("Mortality"[Mesh]) OR ((((((((Mortality[Title/Abstract]) OR (Mortalities[Title/Abstract])) OR (Case Fatality Rate[Title/Abstract])) OR (Case Fatality Rates[Title/Abstract])) OR (Death Rate[Title/Abstract])) OR (Death Rates[Title/Abstract])) OR (Survival[Title/Abstract])) OR (Death[Title/Abstract]))) | 1,365 |

**1.2. Cochrane**

| Search number | Query | Results |
| --- | --- | --- |
| #1 | MeSH descriptor: [Heart Failure] explode all trees | 10671 |
| #2 | (Heart Failure):ti,ab,kw OR (Cardiac Failure):ti,ab,kw OR (Heart Decompensation):ti,ab,kw OR (Myocardial Failure):ti,ab,kw OR (myocardial insufficiency):ti,ab,kw | 45281 |
| #3 | (cardiac backward failure):ti,ab,kw OR (cardiac decompensation):ti,ab,kw OR (cardiac incompetence):ti,ab,kw OR (cardiac insufficiency):ti,ab,kw OR (cardiac stand still):ti,ab,kw | 2074 |
| #4 | (cardial decompensation):ti,ab,kw OR (cardial insufficiency):ti,ab,kw OR (decompensatio cordis):ti,ab,kw OR (heart backward failure):ti,ab,kw OR (heart incompetence):ti,ab,kw | 206 |
| #5 | (heart insufficiency):ti,ab,kw OR (insufficientia cardis):ti,ab,kw | 2853 |
| #6 | #1 or #2 or #3 or #4 or #5 | 46740 |
| #7 | MeSH descriptor: [Machine Learning] explode all trees | 295 |
| #8 | (machine learning):ti,ab,kw OR (Transfer Learning):ti,ab,kw OR (Deep learning):ti,ab,kw OR (Ensemble Learning):ti,ab,kw OR (artificial intelligence):ti,ab,kw | 5596 |
| #9 | (Prediction model):ti,ab,kw OR (random forest):ti,ab,kw OR (neural network):ti,ab,kw OR (neural networks):ti,ab,kw OR (CNN):ti,ab,kw | 8405 |
| #10 | (Support vector machine):ti,ab,kw OR (SVM):ti,ab,kw OR (Gradient Boosting Machine):ti,ab,kw OR (GBM):ti,ab,kw OR (Nomogram):ti,ab,kw | 2861 |
| #11 | (XGBoost):ti,ab,kw OR (Adaboost):ti,ab,kw OR (Decision tree):ti,ab,kw OR (ResNet-50):ti,ab,kw OR (ResNet):ti,ab,kw | 959 |
| #12 | (Fine-Gray):ti,ab,kw OR (Competitive Risk Model):ti,ab,kw OR (Naive Bayesian):ti,ab,kw OR (Risk Prediction):ti,ab,kw OR (Risk-Prediction):ti,ab,kw | 6104 |
| #13 | #7 or #8 or #9 or #10 or #11 or #12 | 18412 |
| #14 | MeSH descriptor: [Mortality] explode all trees | 14102 |
| #15 | (Mortality):ti,ab,kw OR (Mortalities):ti,ab,kw OR (Case Fatality Rate):ti,ab,kw OR (Case Fatality Rates):ti,ab,kw OR (Death Rate):ti,ab,kw | 122747 |
| #16 | (Death Rates):ti,ab,kw OR (Survival):ti,ab,kw OR (Death):ti,ab,kw | 174835 |
| #17 | #14 or #15 or #16 | 231283 |
| #18 | #6 and #13 and #17 | 526 |

**1.3. Embase**

| Search number | Query | Results |
| --- | --- | --- |
| #1 | 'heart failure'/exp | 628293 |
| #2 | 'heart failure':ab,ti OR 'cardiac failure':ab,ti OR 'heart decompensation':ab,ti OR 'myocardial failure':ab,ti OR 'myocardial insufficiency':ab,ti OR 'cardiac backward failure':ab,ti OR 'cardiac decompensation':ab,ti OR 'cardiac incompetence':ab,ti OR 'cardiac insufficiency':ab,ti OR 'cardiac stand still':ab,ti OR 'cardial decompensation':ab,ti OR 'cardial insufficiency':ab,ti OR 'decompensatio cordis':ab,ti OR 'heart backward failure':ab,ti OR 'heart incompetence':ab,ti OR 'heart insufficiency':ab,ti OR 'insufficientia cardis':ab,ti | 357531 |
| #3 | #1 OR #2 | 688497 |
| #4 | 'machine learning'/exp | 359428 |
| #5 | 'machine learning':ab,ti OR 'transfer learning':ab,ti OR 'deep learning':ab,ti OR 'ensemble learning':ab,ti OR 'artificial intelligence':ab,ti OR 'prediction model':ab,ti OR 'random forest':ab,ti OR 'neural network':ab,ti OR 'neural networks':ab,ti OR cnn:ab,ti OR 'support vector machine':ab,ti OR svm:ab,ti OR 'gradient boosting machine':ab,ti OR gbm:ab,ti OR nomogram:ab,ti OR xgboost:ab,ti OR adaboost:ab,ti OR 'decision tree':ab,ti OR 'resnet 50':ab,ti OR resnet:ab,ti OR 'fine gray':ab,ti OR 'competitive risk model':ab,ti OR 'naive bayesian':ab,ti OR 'risk prediction':ab,ti | 334056 |
| #6 | #4 OR #5 | 525770 |
| #7 | 'mortality'/exp | 1337832 |
| #8 | mortality:ab,ti OR mortalities:ab,ti OR 'case fatality rate':ab,ti OR 'case fatality rates':ab,ti OR 'death rate':ab,ti OR 'death rates':ab,ti OR survival:ab,ti OR death:ab,ti | 3603090 |
| #9 | #7 OR #8 | 3926695 |
| #10 | #3 AND #6 AND #9 | 4139 |

**1.4. Web of science**

| Search number | Query | Results |
| --- | --- | --- |
| #1 | "Heart Failure (Topic) OR Cardiac Failure (Topic) OR Heart Decompensation (Topic) OR Myocardial Failure (Topic) OR myocardial insufficiency (Topic) OR cardiac backward failure (Topic) OR cardiac decompensation (Topic) OR cardiac incompetence (Topic) OR cardiac insufficiency (Topic) OR cardiac stand still (Topic) OR cardial decompensation (Topic) OR cardial insufficiency (Topic) OR decompensatio cordis (Topic) OR heart backward failure (Topic) OR heart incompetence (Topic) OR heart insufficiency (Topic) OR insufficientia cardis (Topic) " | 369779 |
| #2 | "machine learning (Topic) OR Transfer Learning (Topic) OR Deep learning (Topic) OR Ensemble Learning (Topic) OR artificial intelligence (Topic) OR Prediction model (Topic) OR random forest (Topic) OR neural network (Topic) OR neural networks (Topic) OR CNN (Topic) OR Support vector machine (Topic) OR SVM (Topic) OR Gradient Boosting Machine (Topic) OR GBM (Topic) OR Nomogram (Topic) OR XGBoost (Topic) OR Adaboost (Topic) OR Decision tree (Topic) OR ResNet-50 (Topic) OR ResNet (Topic) OR Fine-Gray (Topic) OR Competitive Risk Model (Topic) OR Naive Bayesian (Topic) OR Risk Prediction (Topic) OR Risk-Prediction (Topic) " | 2046610 |
| #3 | "Mortality (Topic) OR Mortalities (Topic) OR Case Fatality Rate (Topic) OR Case Fatality Rates (Topic) OR Death Rate (Topic) OR Death Rates (Topic) OR Survival (Topic) OR Death (Topic) " | 3242739 |
| #4 | "#3 AND #2 AND #1 " | 6642 |

Supplemental Table S2: Characteristics and demographics of 28 meta-analyzed studies.

| **Author (year)** | **Country** | **Source of patients** | **Number of death cases** | **Total number of cases** | **Number of death cases in the training set** | **Total number of cases in the training set** | **Generation method of the validation set** | **Method to minimize the overfitting** | **Number of death cases in the validation set** | **Number of cases in the validation set** | **Processing method of missing values** | **Selection method of variables** | **Model types** |
| --- | --- | --- | --- | --- | --- | --- | --- | --- | --- | --- | --- | --- | --- |
| Jie Yang (2022)^14^ | China | Public dataset from India | 333 | 1901 | 333 | 1901 | Internal validation: K-fold cross validation | K-fold cross validation | / | / | Deleted | Single-factor comparison and the weight of evidence | SVM, DT, RF, LightGBM(3) |
| Boshen Yang (2022)^15^ | China | MIMIC-IV, eICU-CRD | MIMIC:395 eICU-CRD: 501 | MIMIC:3210 eICU-CRD: 3862 | 395 | 3210 | Internal validation: K-fold cross validation | K-fold cross validation | / | / | Data with missing values of more than 30% were deleted, and other vacant values were filled by multiple interpolation. | RM algorithm | LR, SVM, DT, Bagging, LightGBM(2), KNN, RF, XGBoost |
| Pengyun Yan (2021)^16^ | China | Single-center | 36 | 489 | 26 | 342 | Internal validation: bootstrap, random sampling method | Bootstrap | 10 | 147 | NA | Univariate and multivariate LR, LASSO | LR |
| Zhe Wang (2020)^17^ | China | Single-center | 539 | 10203 | 539 | 10203 | NA | NA | / | / | NA | Feature rearrangement-based convolutional layer | DL, SVM(2), ANN, LR, RF, XGBoost, LightGBM |
| Zhe Wang (2020)^18^ | China | Single-center | 539 | 4677 patients (10198 records) | 539 | 4677 patients (10198 records) | Internal validation: K-fold cross validation | K-fold cross validation | / | / | NA | Feature selection based on F-value | SVM(2), LR, RF, XGBoost |
| Chuan-he Wang (2021)^19^ | China | Single-center | 307 | 8172 | NA | 4086 | Internal validation: random sampling method | NA | NA | 4086 | Deleted | Univariate and multivariate LR | LR |
| Binhua Wang (2020)^20^ | China | Single-center | 207 | 6182 | 207 | 6182 | Internal validation: K-fold cross validation | K-fold cross validation | / | / | Deleted | Permutation importance algorithm | ANN, SVM(2), LightGBM(2) |
| Yasuyuki Shiraishi (2020)^21^ | Japan | Multicenter | 255 | 6033 | 171 | 4351 | External validation: multicenter | NA | 85 | 1682 | Multiple imputation | Univariate and multivariate LR | LR |
| Matthew W. Segar (2022)^22^ | USA | Multicenter | NA | Training set:123634 Internal validaton:82420 Real world:471086 External validation: 3469 | NA | 123634 | Internal validation: random sampling method; external validation: multicenter | NA | Internal validation: 2351; real world: NA; external validation: 94 | Internal validation: 82420; real world: 471086; external validation: 3469 | Samples with more than 15% missing data were excluded, algorithm interpolation | Random forest-based variable importance selection, LR | LR(2), RF(3) |
| Shengxian Peng (2022)^23^ | China | MIMIC IV, eICU-CRD | 459 | 3458 | 353 | 2766 | Internal validation: random sampling method; external validation: multicenter | NA | 106 | 692 | Algorithm interpolation | LR, LASSO | ANN(2), LR, NB, RF |
| Haichen Lv (2021)^24^ | China | Single-center | 537 (after oversampling: 13065) | 13602 | 90% of 537 (after oversampling 13065) | 90% of 13602 | Internal validation: K-fold cross validation, random sampling method | K-fold cross validation | 10% of 537 (after oversampling 13065) | 10% of 13602 | Variables with missing values greater than 20% were excluded | NA | LR, RF, SVM, ANN, XGBoost |
| Cida Luo (2022)^25^ | China | MIMIC-III, eICU-CRD | MIMIC:595 eICU-CRD: 12.8% of 1349 | MIMIC:5676 eICU-CRD: 1349 | 595 | 5676 | Internal validation: K-fold cross validation; external validation: multicenter | K-fold cross validation | 12.8% of 1349 | 1349 | Covariates with > 40% missing data were excluded, and algorithm interpolation | The permutation-based XGBOOST selection method | LR, XGBoost |
| Le Li (2022)^26^ | China | MIMIC-IV, eICU-CRD | MIMIC:867 eICU-CRD: 533 | MIMIC:4530 eICU-CRD: 2590 | 867 | 4530 | Internal validation: random sampling method; external validation: multicenter | NA | 533 | 2590 | Variables with missing values > 30% were excluded. The multiple  imputation was performed to fill the variables with missing values ≤ 30%. | LASSO | CatBoost, LightGBM, XGBoost, AdaBoost, Bagging, LR, NB, ANN, DT, KNN, SVM |
| Jili Li (2022)^27^ | China | eICU-CRD | 279 | 2798 | 195 | 1958 | Internal validation: random sampling method | NA | 84 | 840 | Multiple imputation | LASSO | XGBoost, LR, RF, SVM |
| Fuhai Li (2021)^28^ | China | MIMIC-III | 159 | 1177 | 116 | 825 | Internal validation: random sampling method | NA | 43 | 352 | Normally distributed continuous variables：mean; skewed distributions continuous variables: median | XGBoost, LASSO, LR | XGBoost, LR |
| Tara Lagu (2018)^29^ | USA | Multicenter | 8910 | 219882 | 80% of 81110 | 80% of 200832 | Internal validation: random sampling method; external validation: multicenter | NA | 800 | 19050 | NA | LR | LR |
| Joon-myoung Kwon (2019)^30^ | Korea | Multicenter | 264 | 6924 | 82 | 2165 | External validation: multicenter | NA | 182 | 4577 | Deleted | NA | DL, RF, LR, SVM, BNN |
| Sebastian König (2021)^31^ | Germany | Multicenter | 3677 | 59125 | NA | 44344 | Internal validation: random sampling method | NA | NA | 14781 | NA | NA | LR, RF, LightGBM, ANN, XGBoost |
| Qian JIA (2017)^32^ | China | Single-center | NA | 2431 | 90 | 1702 | Internal validation: random sampling method | NA | NA | 729 | Deleted | LR | LR |
| Didi Han (2022)^33^ | China | MIMIC IV, eICU-CRD | MIMIC: 12.4% of 15983 eICU-CRD: 12.8% of 14428 | MIMIC:15983 eICU-CRD: 14428 | 12.4% of 15983 | 15983 | External validation: multicenter | NA | 12.8% of 14428 | 14428 | NA | Univariate and multivariate LR | LR |
| Luyao Gao (2021)^34^ | China | Single-center, MIMIC-III | 98 | 226 | 67 | 159 | Internal validation: random sampling method, bootstrap | Bootstrap | 31 | 67 | The proportion of missing values < 5%: mean or median, >5%: multiple imputation, >60%: only the existing data was used | Univariate and multivariate LR | LR |
| Qiying Dai (2022)^35^ | USA | National Inpatient Sample | 106 | 4659 | 81 (after oversampling: 3180) | 3261 | Internal validation: random sampling method | NA | 25 | 1398 | Individuals with missing data on age, gender, race, or in-hospital mortality information were excluded. | LASSO | LR, RF, XGBoost |
| Jiamin Chen (2022)^36^ | China | MIMIC-IV | 707 | 3931 | 707 | 3931 | Internal validation: bootstrap | bootstrap | / | / | Predictive mean matching method | LR, LASSO | LR |
| William T. Abraham (2008)^37^ | USA | Multicenter | 5894 | 220315 | 1217 | 37548 | Internal validation: bootstrap; external validation: multicenter | Bootstrap | OPTIMECHF:28 ADHERE:4649 | OPTIMECHF:937 ADHERE:181830 | Deleted | LR | LR |
| Zijun Chen (2023)^38^ | China | MIMIC-IV, e-ICU-CRD | 2803 | 20993 | 1484 | 11156 | Internal validation: K-fold cross validation; external validation: multicenter | K-fold cross validation | 1319 | 9837 | Multiple imputation | LASSO | XGBoost, LR |
| Meikun Ma (2023)^39^ | China | MIMIC-III | 701 | 4472 | 701 | 4472 | NA | NA | / | / | Algorithm interpolation | NA | LR, RF, SVM, ANN, DL(4) |
| Kayo Misumi (2023)^40^ | Japan | Multicenter | 137 | 2737 | 76 | 1481 | Internal validation: K-fold cross validation; external validation: multicenter | K-fold cross validation | 61 | 1256 | Algorithm interpolation | LASSO | LR |
| Dineo Mpanya1 (2023)^41^ | South Africa | Single-center | 84 | 500 | 59 | 350 | Internal validation: random sampling method | NA | 26 | 150 | The proportion of missing values > 20%: excluded, < 20%: mean or median | NA | RF, LR, SVM, XGBoost, ANN, DT |

AdaBoost, adaptive boosting; ADHERE, Acute Decompensated Heart Failure National Registry; ANN, artificial neural network; BNN, Bayesian network; CatBoost, categorical boosting; DL, deep learning; DT, decision tree; eICU-CRD, Telehealth Intensive Care Unit Collaborative Research Database; KNN, K-nearest neighbors; LASSO, least absolute shrinkage and selection operator; LightGBM, light gradient boosting machine; LR, logistic regression; MIMIC, Medical Information Mart for Intensive Care; NA, not available; NB, Naive Bayes; OPTIME-CHF, Outcomes of a Prospective Trial of Intravenous Milrinone for Exacerbations of Chronic Heart Failure; RF, random forests; SVM, support vector machine; USA, United States of America; XGBoost, eXtreme Gradient Boosting.

**Table S3. The most frequent factors included in risk prediction models for in-hospital death in ICU patients with heart failure.**

| Studies | Age | BUN | Urine output | Mechanical ventilation (Respiratory support) | NT-proBNP | Respiratory rate | Acute kidney injury | HR | SpO2 | Calcium |
| --- | --- | --- | --- | --- | --- | --- | --- | --- | --- | --- |
| Boshen Yang^15^ | 9 | 9 | 9 |  | 9 | 9 |  | 9 |  |  |
| Shengxian Peng^23^ | 5 | 5 |  | 5 | 5 | 5 |  | 5 | 5 | 5 |
| Cida Luo^25^ | 2 | 2 | 2 |  |  | 2 |  |  | 2 | 2 |
| Le Li^26^ | 11 |  | 11 | 11 | 11 |  | 11 |  | 11 |  |
| Jili Li^27^ | 4 | 4 | 4 |  |  | 4 | 4 | 4 | 4 | 4 |
| Fuhai Li^28^ | 2 | 2 | 2 |  | 2 | 2 |  | 2 |  | 2 |
| Didi Han^33^ | 1 | 1 |  | 1 |  | 1 |  | 1 |  |  |
| Luyao Gao^34^ | 1 |  |  |  |  |  |  |  |  |  |
| Jiamin Chen^36^ | 1 |  |  |  |  |  | 1 | 1 |  |  |
| Zijun Chen^38^ | 2 |  |  | 2 |  | 2 |  |  |  |  |
| Meikun Ma^39^ |  | 8 |  | 8 |  |  | 8 |  |  | 8 |
| Total | 38 | 31 | 28 | 27 | 27 | 25 | 24 | 22 | 22 | 21 |

BUN, blood urea nitrogen; HR, heart rate; ICU, intensive care unit; NT-proBNP, N-terminal pro-B-type natriuretic peptide; SpO2, blood Oxygen saturation.
